# Antiosteoclastic Activity of Milk Thistle Extract after Ovariectomy to Suppress Estrogen Deficiency-Induced Osteoporosis

**DOI:** 10.1155/2013/919374

**Published:** 2013-05-28

**Authors:** Jung-Lye Kim, Yun-Ho Kim, Min-Kyung Kang, Ju-Hyun Gong, Seoung-Jun Han, Young-Hee Kang

**Affiliations:** ^1^Department of Food and Nutrition, Hallym University, Chuncheon, Kangwon-do 200-702, Republic of Korea; ^2^Seorim Bio, Chuncheon, Kangwon-do 200-944, Republic of Korea

## Abstract

Bone integrity abnormality and imbalance between bone formation by osteoblasts and bone resorption by osteoclasts are known to result in metabolic bone diseases such as osteoporosis. Silymarin-rich milk thistle extract (MTE) and its component silibinin enhanced alkaline phosphatase activity of osteoblasts but reduced tartrate-resistant acid phosphatase (TRAP) activity of osteoclasts. The osteoprotective effects of MTE were comparable to those of estrogenic isoflavone. Low-dose combination of MTE and isoflavone had a pharmacological synergy that may be useful for osteogenic activity. This study attempted to reveal the suppressive effects of MTE on bone loss. C57BL/6 female mice were ovariectomized (OVX) as a model for postmenopausal osteopenia and orally administered 10 mg/kg MTE or silibinin for 8 weeks. The sham-operated mice served as estrogen controls. The treatment of ovariectomized mice with nontoxic MTE and silibinin improved femoral bone mineral density and serum receptor activator of nuclear factor-**κ**B ligand/osteoprotegerin ratio, an index of osteoclastogenic stimulus. In addition, the administration of MTE or silibinin inhibited femoral bone loss induced by ovariectomy and suppressed femoral TRAP activity and cathepsin K induction responsible for osteoclastogenesis and bone resorption. Collectively, oral dosage of MTE containing silibinin in the preclinical setting is effective in preventing estrogen deficiency-induced bone loss.

## 1. Introduction

Osteoporosis is a common metabolic bone-related disease characterized by low bone mass and microarchitectural deformation of bone tissue leading to increased bone fragility and fractures [1]. Primary type 1 osteoporosis or postmenopausal osteoporosis takes place most commonly in women after menopause [2]. Primary type 2 osteoporosis or senile osteoporosis is seen in both females and males after age 75. In postmenopausal osteoporosis estrogen deficiencies lead to high bone turnover and bone loss [2, 3]. Osteoporosis risks can be reduced with lifestyle changes such as diet and exercise and sometimes medication. Antiosteoporotic medication on fracture healing includes calcium, vitamin D, bisphosphonates, denosumab, and several others [3, 4]. Hormone replacement therapy has been one of the most effective treatments for the prevention of adverse postmenopausal changes [5]. Current pharmacological therapies for osteoporosis include agents that either inhibit bone resorption or stimulate bone formation, thereby leading to more efficient recovery of bone mass in osteoporosis [6].

There is a need to develop harmless and affordable alternative therapies for preventing osteoporosis. The use of complementary therapies to alleviate postmenopausal osteoporosis is fairly widespread among women. Natural products and dietary components have positive effects on bone remodeling, particularly by inhibiting bone resorption [7, 8]. Studies in humans and animals have been focused on accumulating the biological potency of phytoestrogens, as well as on revealing the mechanism(s) by which these plant compounds prevent bone loss [9, 10]. Phytoestrogens with estrogen-like biological activity include isoflavones, prenylated flavonoids, coumestans, and lignans. Phytoestrogens mainly belong to naturally occurring nonsteroidal plant compounds that, because of their structural similarity with 17*β*-estradiol, have the ability to cause estrogenic or/and antiestrogenic effects primarily through binding to estrogen receptors (ER) [11]. The isoflavone daidzein prevents bone loss by reversing the detrimental immune changes as a result of estrogen deficiency as a key mechanism for antiosteoclastogenic effect [12]. It has been reported that resveratrol as a naturally occurring phytoestrogen possesses bone-protective effects by antagonizing adipogenesis [13]. 

To identify new, naturally occurring antiosteoporotic agents, estrogen-like compounds widely used in traditional medicine were screened for their inhibitory activity in the bone resorption. Resveratrol promotes osteogenesis of human mesenchymal stem cells by upregulating RUNX2 gene expression via the SIRT1/FOXO3A axis as a novel mechanism [13]. Our previous study found that silymarin rich in milk thistle exerted osteoblastic activity in osteoblasts and tibia-fractured mice [14]. In fact, silymarin, the isomeric mixture of flavonolignans extracted from milk thistle (Silybum marianum), comprises silibinin A and B, isosilibinin A and B, silychristin, and sildianin. Silibinin prevents memory impairment and oxidative damage induced by amyloid beta peptide [15]. Additionally, oral supplementation of silibinin prevents colon carcinogenesis [16]. We demonstrated that the flavonolignan silibinin, the major active constituent of silymarin, enhanced osteoblastogenesis and osteoprotection in osteoblasts and osteoclasts [17]. However, the action mechanisms of silibinin for modulating bone-remodeling process still remain unclear under in vivo conditions. 

This study attempted to determine whether silymarin-rich milk thistle *silybum marianum L. Gaertn* extract (MTE) would help to prevent osteoporosis associated with estrogens deficiency. This study evaluated the effects of MTE on parameters and histological status of femoral bone and expression of bone-specific genes in ovariectomized mice. The sham-operated mice were fed the control diet with and OVX mice were daily fed with control diets or diets containing 10 mg/kg MTE or 10 mg/kg silibinin for 8 weeks. MTE prevented bone loss induced by estrogen deficiency through promoting osteoblastogenesis and inhibiting osteoclastogenesis of the MTE component silibinin. Therefore, MTE rich in silibinin would be a potential alternative treatment for prevention of postmenopausal osteoporosis. Low-dose combination of MTE and isoflavone had a pharmacological synergy that may be useful for osteogenic activity.

## 2. Materials and Methods

### 2.1. Materials

Fetal bovine serum (FBS), penicillin-streptomycin, and trypsin-EDTA were purchased from Lonza (Walkersville, MD). 3-(4,5-Dimethylthiazol-yl)-diphenyl tetrazolium bromide (MTT) was provided by DUCHEFA Biochemie (Haarlem, Netherlands). Minimum essential medium alpha medium (*α*-MEM), Dulbecco's modified eagle's media (DMEM), receptor activator of nuclear factor-*κ*B ligand (RANKL), silymarin, and silibinin were supplied by Sigma-Aldrich Chemicals (St. Louis, MO), as were all other reagents, unless specifically stated elsewhere. Antimouse cathepsin K was purchased from Santa Cruz Biotechnology (Santa Cruz, CA). Horseradish peroxidase-conjugated goat antirabbit IgG was obtained from Jackson ImmunoResearch Laboratories (West Grove, PA). Milk thistle, silibinin, and isoflavone were dissolved in dimethyl sulfoxide (DMSO) for live culture with cells; a final culture concentration of DMSO was <0.5%.

### 2.2. Preparation of MTE and HPLC Analyses

Milk thistle was pulverized and extracted in 95% ethanol, and the obtained extract was decanted and filtered to give a crude extract. The crude extract was evaporated under reduced pressure to dryness. HPLC analysis of MTE was performed at ambient temperature using a HPLC system (SHIMADZU, Kyoto, Japan) with a Waters Bondapak C18 (3.9 × 300 mm) column. The mobile phases were a binary elution of water : methanol : H_3_PO_4_ (400 : 100 : 2.5) and water : methanol : H_3_PO_4_ (100 : 400 : 2.5) and were degassed ultrasonically prior to use. The column was thermostated at 40°C, gradient elution mode at the flow rate of 1 mL/min was used and injection volume was 10 *μ*L. Eluted substances were detected at 288 nm with a photodiode-array detector (PAD). Silymarin and silibinin were used for the calibration standards. Total contents (%) of silymarin and silibinin in MTE were calculated as follows: silymarin or silibinin total content = (sample area/standard area)∗(standard weight/sample weight)∗100.

HPLC analysis of prepared soybean extract (SBE) was also performed at ambient temperature using a HPLC system with a Mitsubishi DIAION HP-20 column. The mobile phases were a binary elution of water : methanol : H_3_PO_4_ (88 : 10 : 2) and methanol : H_3_PO_4_ (98 : 2) and were degassed prior to use. Eluted substances were detected at *λ* = 260 nm with a PDA. Daidzin, genistin, glycitin, daidzein, genistein, and glycitein were used for the calibration standards. Total contents of isoflavone in SBE were calculated as follows: isoflavone (aglycones of daidzein, genistein, and glycitein) contents = sample concentration (*μ*g/mL) × [sample total volume (mL)/extract volume (g)]∗standard purity∗(1/1,000). The conversion ratio (1/1.6 = 0.625) was applied for the contents of glycoside.

### 2.3. Culture of Osteoblasts and Osteoclasts

MC3T3-E1 cells (mouse calvaria origin) were cultured at 37°C in a humidified atmosphere of 5% CO_2_ in air. MC3T3-E1 was cultured in *α*-MEM containing 10% FBS, 2 mM glutamine, 100 U/mL penicillin, and 100 *μ*g/mL streptomycin. For the osteoblast differentiation, MC3T3-E1 cells were seeded on 24-well plates and grown to *≈*90% confluence, and then the culture medium was changed to a fresh normal osteogenic medium containing 10 mM *β*-glycerolphosphate and 50 *μ*g/mL ascorbic acid in the presence of MTE, silibinin and/or SBE to initiate matrix maturation. Cell culture medium was changed every 3 days for 15 days. 

Murine macrophage RAW264.7 cells were cultured in DMEM containing 10% FBS, 2 mM glutamine, 100 U/mL penicillin, and 100 *μ*g/mL streptomycin at 37°C in a humidified atmosphere of 5% CO_2_ in air. For the osteoclast differentiation, cells were seeded on 24-well plates and grown to *≈*90% confluence in *α*-MEM containing 50 ng/mL receptor activator of nuclear factor-*κ*B ligand (RANKL) for 5 days. Cell culture media were changed every 2 days for 5 days. 

### 2.4. Animals and Ovariectomy

To determine inhibitory effects of MTE and silibinin on osteoporotic activity in estrogen-deficient animal model, this study employed ovariectomy technique for mimicking estrogen deprivation or senescent menopause. C57BL/6 mice (11 weeks of age, 20–25 g) were obtained from the Experimental Animal Center, Hallym University, and kept on a 12-hour light/dark cycle at 20–25°C with 60% relative humidity under specific pathogen-free conditions. Mice were fed with a nonpurified diet (RodFeed, DBL, Samsung, Korea) during the experimental period (8 weeks) with free access to water *ad libitum* at the animal facility of Hallym University. The animals were allowed to acclimatize for a week before beginning experiments. All animal experiments were performed in accordance with the University's Guidelines for the Care and Use of Laboratory Animals approved by the Committee on Animal Experimentation of Hallym University (permission number: Hallym 2011-24).

For the ovariectomical surgery [18], 11-week-old female animals were anesthetized using a ketamine/Rompun cocktail (40 mg ketamine and 10 mg rompun/kg body weight) for either a sham operation (Sham) or bilateral oophorectomy (ovariectomy, OVX). Mice receiving surgical OVX were orally treated with 10 mg/kg MTE or 10 mg/kg silibinin once a day for 8 weeks (9 mice of each group). After 8 weeks of treatment, blood samples and uterus tissues were collected, and serum samples obtained by centrifugation (3,000 rpm, 10 min) were stored at −70°C prior to analyses. 

After 8 weeks following ovariectomical surgery, the final body weight (BW) and average daily gain (ADG) significantly increased in OVX mice as compared to sham-operated control mice (Table 1). A tendency toward decrement for final BW and ADG in OVX mice supplemented with MTE and silibinin was observed. No significant difference was observed in the average daily feed intake (ADFI) of each mouse group. However, the food efficiency ratio (FER = ADG/ADFI) was significantly higher than that of the other groups (Table 1). Ovariectomy reduced the liver weight-to-BW ratio (LW/BW) as compared to sham-operated mice. 

### 2.5. Activity Measurement of Alkaline Phosphatase (ALP) and Tartrate-Resistant Acid Phosphatase (TRAP)

ALP activity of MC3T3-E1 osteoblasts was determined by a modified colorimetric enzyme assay [19]. After culture protocols, cells were lysed with 0.2% Triton X-100, and the lysates were centrifuged at 14,000 ×g for 10 min at 4°C. Lysate aliquots were incubated with 0.5 M Tris-HCl buffer (pH 9.9) containing 6 mM *p*-nitrophenyl phosphate and 1 mM MgCl_2_ at 37°C for 1 h. ALP activity was expressed as nmol *p*-nitrophenol (PNP) produced/min/mg protein. Absorbance was measured at *λ* = 405 nm by comparing with PNP standards.

For the measurement of TRAP activity, cells were fixed with 4% formalin solution for 10 min and 95% ethanol for 1 min. Subsequently, the dried cells were incubated in 10 mM citrate buffer (pH 4.6) containing 10 mM sodium tartrate and 5 mM *p*-nitrophenylphosphate. After incubation for 1 h, the reaction mixtures were transferred to new well plates containing an equal volume of 0.1 N NaOH. Absorbance was measured at *λ* = 405 nm by spectrophotometer, and the TRAP activity was expressed as percent of that of RANKL-untreated control.

### 2.6. Serum Biochemical Analyses

Serum 17*β*-estradiol levels were determined by using enzyme-linked immunosorbent assay (ELISA) kits (Uscn Life Science, Wuhan, China) according to the manufacturer's instructions. Serum levels of osteoprotegerin (OPG) and RANKL were also determined by using ELISA assay kits (R&D Systems, Minneapolis, MN).

Serum levels of glutamic oxaloacetic transaminase (GOT) and glutamic pyruvic transaminase (GPT) were measured by using enzymatic kits (Asan Pharmaceuticals, Seoul, Korea) according to the manufacturer's instructions. In this study, serum GOT and GPT levels were not influenced by the administration of MTE and silibinin to OVX mice, indicating that they did not induce liver toxicity (Table 1).

### 2.7. Assessment of Bone Mineral Density (BMD)

BMD and bone mineral content (BMC) of mouse femoral bone were determined with a PIXImus mouse densitometer (GE Lunar, Waukesha, WI, USA). BMD calculated by dividing BMC (mg) by the projected bone area (cm^2^) was assessed in the femoral regions. 

### 2.8. Histological Examination and TRAP Staining

Uterus and femoral bone tissues were obtained from three mice of each group. After being washed with saline, uterine tissues were fixed in 10% neutral *buffered* formalin for 24 h. Tissues were stained using a modified Harris hematoxylin and Shandon Instant eosin (H&E) for the microscopic observation. 

Femoral bone tissues were decalcified in decalcifying solution (Sigma-Aldrich Chemicals) and dehydrated in a graded series of ethanol solutions for 18 h. For the histological staining of H&E and TRAP, femoral bone tissues were then embedded in paraffin and cut into 5 *μ*m sections in thickness. TRAP staining was conducted by using leukocyte acid phosphatase kit (Sigma Chemicals), according to the manufacturer's instructions. Femoral tissue samples were incubated for 20 min in sodium acetate (50 mM) and potassium sodium tartrate (40 mM) buffer (pH 5.0) and then incubated for further 15 min at room temperature in the same buffer containing naphthol AS-BI phosphate (2.5 mg/mL) and fast garnet GBC (0.5 mg/mL). After each slide was mounted in VectaMount mounting medium (Vector Laboratories, Burlingame, CA), images were taken using an Axiomager optical microscope system (Zeiss, Germany). TRAP activity was quantified by image analysis program of the microscope system. 

### 2.9. Immunohistochemical Analysis

Immunohistochemical analysis was carried out by using an antibody against mouse cathepsin K. Femoral bone tissue sections were subject to a series of immunohistochemical procedures including the Ag retrieval followed by quenching of endogenous peroxidase activity. For the measurement of bone tissue level of cathepsin K, an immunohistochemical analysis was conducted using 3,3′-diaminobenzidine chromogenic substrate detection kit (Dako, Carpinteria, CA). Counter staining was conducted with hematoxylin. Each slide was mounted in VectaMount mounting medium (Vector Laboratories). Images of each slide were taken using an optical microscope system (Axiomager). Bone tissue protein level of cathepsin K was quantified by the image analysis program of the microscope system. 

### 2.10. Statistical Analysis

The data are presented as mean ± SEM. Statistical analyses were carried out using Statistical Analysis System statistical software package (SAS Institute Inc, Cary, NC). Significance was determined by one-way analysis of variance, followed by the Duncan range test for multiple comparisons. Differences were considered significant at *P* < 0.05.

## 3. Results

### 3.1. Identification of MTE

**Figure 1:**
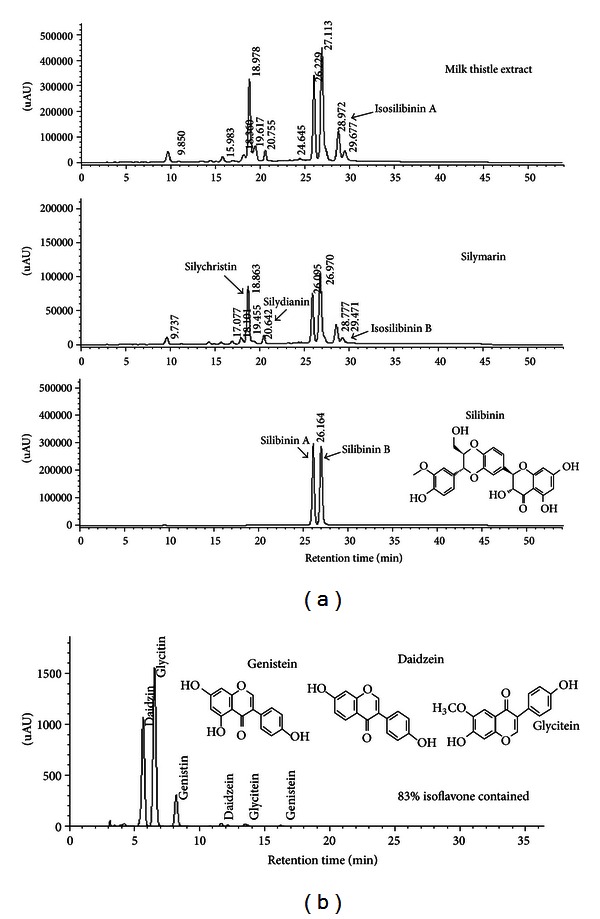
HPLC spectra for chemical constituents of milk thistle extract, silymarin, and silibinin (chemical structure) (a) and for isoflavones present in prepared soybean extract (b). The peaks of silymarin and silibinin were identified, as confirmed by the UV contour plot obtained by a photodiode-array detector (a). The peaks of daidzin, genistin, glycitin, daidzein, genistein, and glycitein were also identified (b).

The HPLC spectra of MTE obtained at *λ* = 288 nm were nearly identical to those of silymarin with several different peaks, indicating that there were several compounds present (Figure 1). Three distinct peaks were detected in MTE with different retention times, in which two peaks were identified to be silibinin A, and silibinin B, as confirmed by the HPLC spectra of silibinin. It has been shown that silymarin contains taxifolin, silychristin, silydianin, silibinin A, silibinin B, isosilibinin A and isosilibinin B in different amounts [20, 21]. Consistent with a previous report [22], the peak retained at 18.9 min was identified as silychristin (Figure 1). When the contents of the silymarin were calculated from peak area and weight measurements, silymarin accounted for 80.04% of all compounds present in MTE. Based on the ratio of the peak area as determined by HPLC, the purity of silibinin was 31.37% in an industrial scale. 

Total content of isoflavone in prepared SBE was 83%, containing daidzin, genistin, glycitin, daidzein, genistein, and glycitein detected as 6 different peaks (Figure 1(b)). 

### 3.2. Modulation of Osteoblastic ALP Activity and Osteoclastic TRAP Activity by MTE

**Figure 2:**
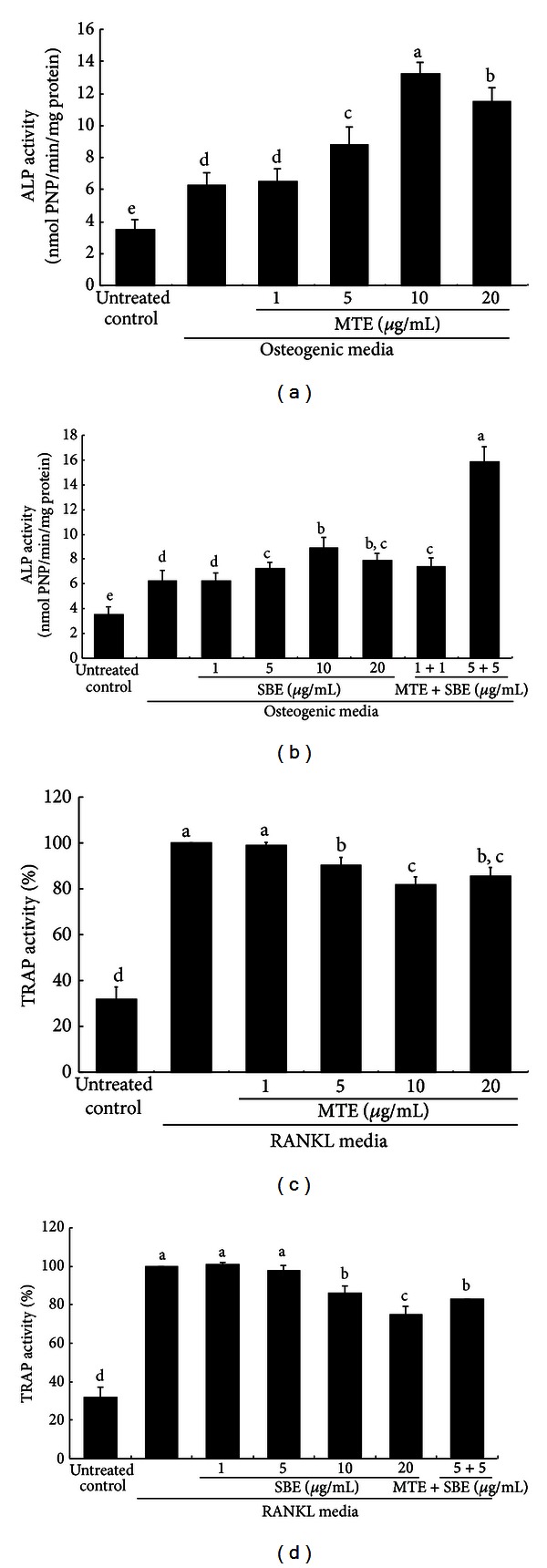
Effects of milk thistle extract (MTE) and soybean extract (SBE) on alkaline phosphatase (ALP) activity of osteoblasts ((a) and (b)) and tartrate-resistant acid phosphatase (TRAP) activity of osteoclasts ((c) and (d)). After differentiation of MC3T3-E1 cells to osteoblasts and of RAW264.7 cells to osteoclasts, MTE ((a) and (c)) and SBE ((b) and (d)) were treated individually or in combination at the doses indicated. To detect activity of ALP in MC3T3-E1 cell media on day 6, a colorimetric enzyme assay was employed. Absorbance was measured at *λ* = 405 nm and compared with *p-*nitrophenol (PNP) standard (mean ± SEM, *n* = 4). The ALP enzyme activity was expressed as nmol PNP/min/mg protein. For the measurement of TRAP activity, RAW 264.7 cells were exposed to 50 ng/mL RANKL for 5 days. After 5-day culture, TRAP-positive activity was determined at *λ* = 405 nm (mean ± SEM, *n* = 3). Values in bar graphs not sharing a letter indicate significant difference at *P* < 0.05.

Since ALP is a biomarker for the matrix maturation of osteoblasts, this study investigated the osteoblastic activity of MTE. The cellular ALP activity increased during 6-day differentiation in normal osteogenic medium, which was positively influenced by treatment with ≥5 *μ*g/mL MTE or ≥10 *μ*g/mL SBE (Figures 2(a) and 2(b)). Additionally, the low-dose combination of MTE and SBE had a synergistic mechanism of action that may be useful for osteoblastic activity (Figure 2(b)). Furthermore, the current study attempted to examine osteoclastic TRAP activity in differentiated RAW 264.7 cells. Stimulation with RANKL for 5 days differentiated RAW 264.7 macrophages to TRAP-positive multinucleated cells. When cells were incubated with 1–20 *μ*g/mL MTE and SBE, the TRAP activity was notably diminished (Figures 2(c) and 2(d)). Pharmacological synergy between MTE and SBE offered advantages in the treatment of osteoporosis by using lower individual dosages (Figure 2(d)).

### 3.3. Improvement of Uterine Size and Serum 17*β*-Estradiol Level by MTE

**Figure 3:**
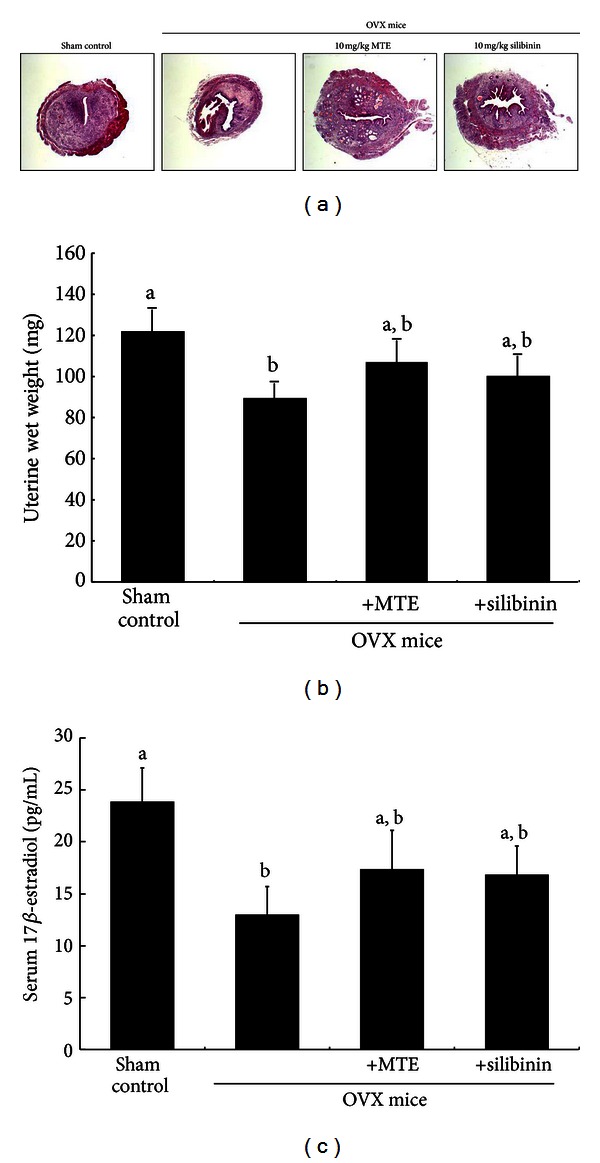
Uterus transverse section (a), wet weight of uterine tissue (b), and serum 17*β*-estradiol level (c) of ovariectomized (OVX) mice treated with milk thistle extract (MTE) and silibinin. OVX mice were orally treated with 10 mg/kg/day MTE or 10 mg/kg/day silibinin daily for 8 weeks. After being washed with saline, uterine tissues were fixed in 10% neutral buffered formalin for 24 h. Cross-sectional images of uterine horn were obtained by staining with hematoxylin and eosin and visualized under light microscopy (3 separate experiments). Magnification: 40-fold. Serum 17*β*-estradiol level was determined by using assay kits. Values in bar graphs (mean ± SEM, *n* = 9) not sharing a letter indicate significant difference at *P* < 0.05.

Estrogen loss surgically induced for 8 weeks caused a marked atrophy of the uterus. There was a marked reduction of the uterus in size and wet weight due to OVX (Figures 3(a) and 3(b)). Administration of MTE and silibinin to OVX mice nearly restored the uterus to its normal size. MTE and silibinin tended to increase the wet weight of OVX mouse uterine (Figure 3(b)). As expected, the serum 17*β*-estradiol level declined in OVX mice by half (Figure 3(c)). However, the administration of MTE and silibinin appeared to enhance its level to some extent. 

### 3.4. Effect of MTE on Ovariectomy-Induced Bone Loss

Significant loss of femoral BMD in mice was observed at 8 weeks after OVX (Table 2). However, the treatment of OVX mice with either MTE or silibinin resulted in a significant increase in BMD and BMC at 8 weeks after OVX. The increment in BMD and BMC was much higher in MTE-treated OVX mice. The BMD and BMC were nearly restored by supplementing 10 mg/kg MTE, as compared to those of sham-operated control mice (Table 2). 

**Figure 4:**
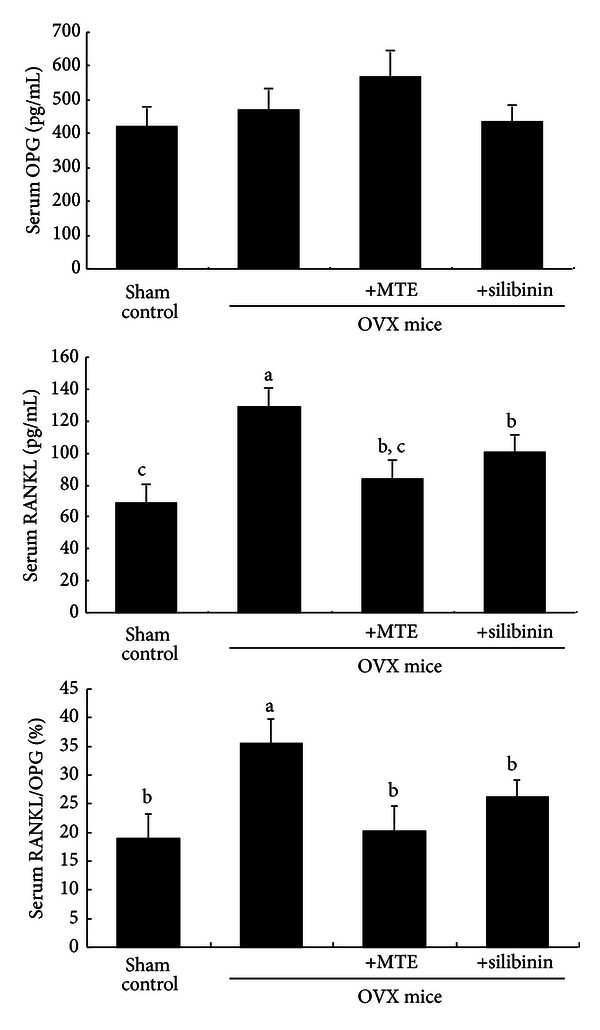
Suppressive effects of milk thistle extract (MTE) and silibinin on serum RANKL/OPG ratio in ovariectomized (OVX) mice. OVX mice were orally administrated with 10 mg/kg/day MTE or 10 mg/kg/day silibinin daily for 8 weeks. Serum levels of OPG and RANKL were determined by using ELISA kits. Values in bar graphs (mean ± SEM, *n* = 9) not sharing a letter indicate significant difference at *P* < 0.05.

Serum levels of OPG and RANKL were assessed on the 8th week following surgical estrogen deprivation. Serum OPG level was not significantly influenced by uterine loss, whereas serum RANKL level was enhanced in OVX mice, as compared to that of sham-operated mice (Figure 4). When MTE or silibinin was treated for 8 weeks after OVX, the serum RANKL level was reduced. Thus, the ratio of serum RANKL and OPG decreased in OVX mice treated with MTE or silibinin (Figure 4). 

### 3.5. Inhibition of Ovariectomy-Induced Osteoclastic Activity by MTE

**Figure 5:**
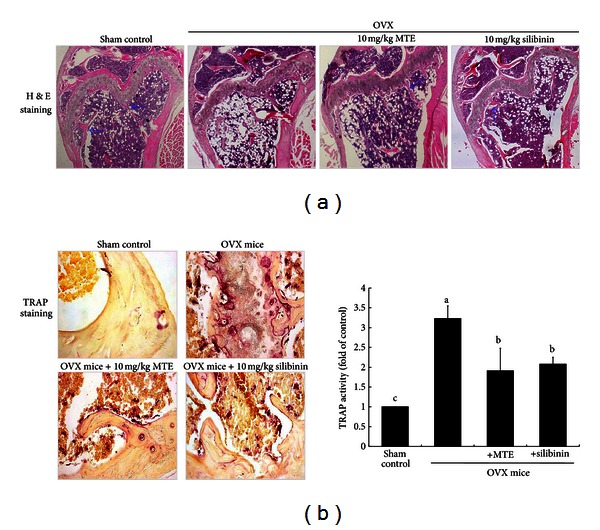
Histological femoral morphology (distal portions of the femora, (a)) and TRAP localization (b) in femoral bone tissue sections of ovariectomized (OVX) mice administrated with milk thistle extract (MTE) and silibinin. OVX mice were orally administrated with 10 mg/kg/day MTE or 10 mg/kg/day silibinin daily for 8 weeks. Longitudinal femoral bone tissues were stained using hematoxylin and eosin (H&E) for the microscopic observation (a). The arrows indicate trabecular bones in the metaphysis and diaphysis. TRAP staining of cross-sections of femoral bone (b) was conducted by using leukocyte acid phosphatase kits, and the staining intensity (mean ± SEM, *n* = 3) was measured. TRAP-positive osteoclasts (purple) are observed at the termini of metaphyseal trabeculae. Values in bar graphs not sharing a letter indicate significant difference at *P* < 0.05. Representative images were visualized under light microsopy (3 separate experiments). Magnification: 40-fold.

In the 8th week after ovariectomy, the histological sagittal sections of the femora showed that the bony meshwork appeared in both the metaphysis and diaphysis of OVX mice when compared with the sham-operated group (Figure 5(a)). There was apparent reduction in trabecular (cancellous) bones in OVX group. In contrast, the spongy trabecular bones appeared to be added in the metaphyseal area of OVX mice treated with MTE or silibinin. Cancellous bones are one of types of osseous tissues that form bones. Consistent with the histological findings, there was a significant increase in BMD in the femur bones of MTE-treated mice compared with those of OVX-alone group (Table 2). 

The TRAP staining showed larger osteoclasts (purple-stained) in OVX mouse femora compared with those in the sham-operated control mice (Figure 5(b)). At 8 weeks after OVX, many TRAP-positive osteoclasts were observed at the termini of metaphyseal trabeculae of OVX mice, while TRAP activity of osteoclasts in the MTE-treated mice was similar to that of the sham group. There was a similar reduction in TRAP activity detected in silibinin-treated mice. Therefore, MTE as well as silibinin appeared to lessen the increase in osteoclastic bone resorption induced by OVX. 

**Figure 6:**
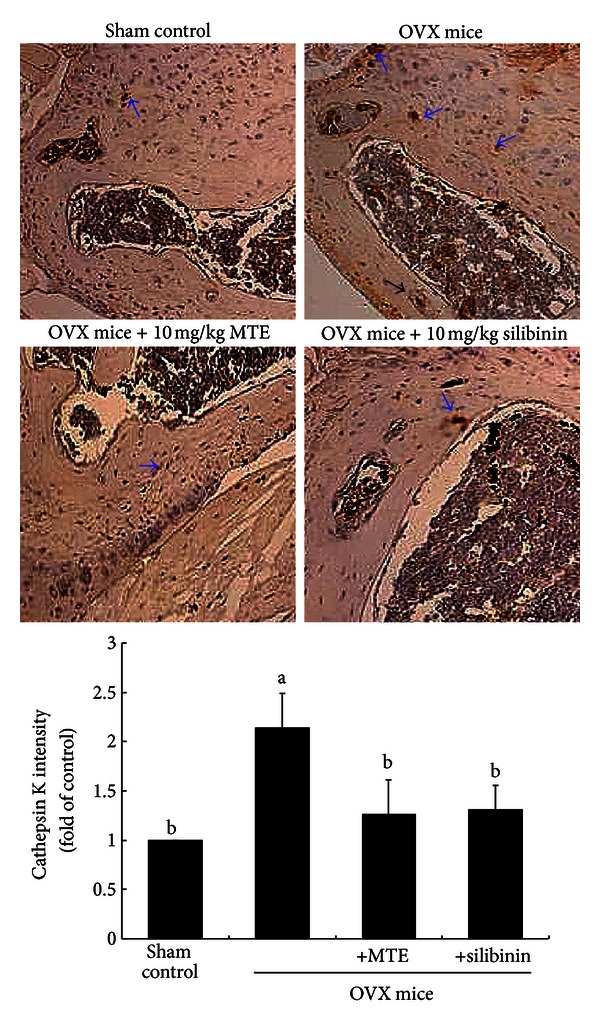
Immunohistochemical analysis showing induction of cathepsin K in femoral bone tissue sections of ovariectomized (OVX) mice administrated with milk thistle extract (MTE) and silibinin. OVX mice were orally administrated with 10 mg/kg/day MTE or 10 mg/kg/day silibinin daily for 8 weeks. Immunohistochemical staining was conducted using a primary antibody of mouse cathepsin K (brown staining, arrows). Representative images were visualized under light microscopy, and brown staining intensity was measured (3 separate experiments). Magnification: 40-fold. Staining intensity values (mean ± SEM, *n* = 3) in bar graphs not sharing a letter indicate significant difference at *P* < 0.05.

This study further investigated whether MTE attenuated cathepsin K induction in OVX-challenged mice. It is well known that cathepsin K is the protease that is primarily responsible for the degradation of bone matrix by osteoclasts. The inhibition of cathepsin K is related to sustained reductions in bone resorption markers but with minimal and transient reductions in bone formation markers [23]. Oral administration of 10 mg/kg MTE or silibinin suppressed the cathepsin K (arrows) induction enhanced in OVX mouse femur bones, evidenced by immunohistochemical 3,3′-diaminobenzidine staining (Figure 6). Likewise, the attenuation of cathepsin K induction was notably observed in femoral tissues of OVX-challenged mice orally treated with 10 mg/kg silibinin, indicating antiosteoclastic activity of MTE or silibinin. 

## 4. Discussion

Six major findings were extracted from this study. (1) MTE promoted bone-forming activity of osteoblasts and inhibited bone-degrading activity of osteoclasts, which was attributed to osteoprotective effects of silibinin, major constituent of MTE. (2) Pharmacological synergy between MTE and isoflavone-containing SBE offered advantages in the treatment of osteoporosis by using lower individual doses. (3) The levels of BMD and BMC diminished in OVX mice were enhanced and nearly restored by supplementing 10 mg/kg MTE or silibinin, as compared to those of sham-operated control mice. (4) MTE was an inhibitor dampening serum RANKL elevated due to estrogen deficiency. (5) Cancellous bones appeared to be trabecular in the metaphysis and diaphysis of OVX mouse femora treated with MTE or silibinin. (6) MTE and silibinin inhibited osteoclastic bone resorption through suppressing TRAP activity and cathepsin K induction in OVX mouse femora. Therefore, silibinin-containing MTE prevented bone loss induced by estrogen deficiency through promoting osteoblastogenesis and encumbering osteoclastogenesis. MTE rich in silibinin would be a potential alternative treatment for prevention of postmenopausal osteoporosis.

 Osteoporosis is a degenerative bone disease caused by low BMD and microarchitectural deterioration, resulting increased risk of fracture with bone weakness [1]. Postmenopausal osteoporosis takes place due to estrogen deficiency resulting in high bone turnover and bone loss [2, 3]. Estrogen deficiency plays a causative role in the activity of osteoclasts. Several antiosteoporotic medications including calcium and vitamin D have been used to heal or alleviate fracture and osteoporosis [3, 4]. Hormone therapy has been one of the most effective initiations around the time of menopause to treat menopause-related symptoms and to prevent osteoporosis in women at high risk of fracture [5, 24]. Bisphosphonates are a class of drugs used to treat osteoporosis and related diseases by inhibiting bone digestion of osteoclasts and slowing bone loss [24]. Denosumab is a human monoclonal antibody designed to target RANKL for the treatment of osteoporosis bone metastases and rheumatoid arthritis [25]. RANKL is a protein that acts as the primary signal for bone removal and accelerates bone destruction. Current pharmacological therapies for osteoporosis include agents that either inhibit bone resorption or stimulate bone formation, thereby leading to more efficient recovery of bone mass in osteoporosis [6]. In current study, silymarin-rich MTE and silibinin inhibited RANKL-induced bone-degrading activity of osteoclasts. In addition, the bone-forming ALP activity of osteoblasts was also enhanced by MTE. Therefore, MTE may be a therapeutic agent promoting matrix mineralization and antagonizing bone loss.

The use of complementary therapies to alleviate osteoporosis is fairly widespread among women. It is of significance to develop natural alternative therapies for preventing postmenopausal or senile osteoporosis. Numerous studies have shown that natural products and dietary components such as phytoestrogens have positive effects on bone remodeling particularly by inhibiting bone resorption [7, 8]. The action mechanism(s) by which these plant compounds prevent bone loss have been reported [9, 10]. Phytoestrogens with estrogen-like biological activity include isoflavones, prenylated flavonoids, coumestans, and lignans, which have a similar structure to 17*β*-estradiol and bind to ER [11]. Soy isoflavone derivatives increase BMD from mice with postmenopausal estrogen deficiency [26]. Lignan-rich flaxseed combined low-dose estrogen therapy greatly protects against ovariectomy-induced bone loss at the lumbar vertebrae [27]. In fact, this study found that the combination of low-dose MTE and isoflavone showed a synergic effect on osteogenic and osteoprotective activity of osteoblasts and osteoclasts. Since MTE showed osteoblastogenic and osteoclastogenic activity, this study further attempted to determine whether silymarin-rich MTE and silibinin inhibited osteoporosis associated with estrogens deficiency.

Complementary approaches such as the use of natural food ingredients for the prevention and management of postmenopausal osteoporosis are worth exploring. Milk thistle has been clinically used for its beneficial effects on various liver diseases such as alcohol or drug intoxication and viral hepatitis [28]. Silymarin is a constitutive flavonolignans composed of silychristin, isosilychristin, silydianin, silibinin, and isosilibinin [21]. Our recent study demonstrated that silymarin ameliorated bone healing through improving tibial bone strength with elevated BMD and serum levels of osteogenic ALP and osteocalcin in tibia-fractured mice [14]. However, the action mechanisms of silymarin for promoting bone healing process still remain unclear. This study evaluated how MTE influenced histological status of femoral bones, and bone-specific gene induction occurred after uterine deprivation. MTE prevented postmenopausal bone loss induced by estrogen deficiency through inhibiting RANKL production of OVX mice as a RANKL inhibitor. RANKL promotes the maturation of osteoclasts, and OPG prevents from binding of RANKL to RANK receptor on osteoclasts resulting in inhibition of osteoclast differentiation [29]. Silibinin-containing MTE administration diminished serum RANKL/OPG ratio of OVX mice, alleviating osteoclastogenesis enhanced after estrogen deficiency. Additionally, MTE suppressed TRAP activity and cathepsin K induction enhanced in the OVX femur, indicating the numeral reduction of osteoclasts causing bone resorption. These findings revealed that silibinin rich in MTE as glycoside may prevent postmenopausal osteoporosis due to estrogen deficiency through dampening osteoclastogenesis. 

In summary, the current report demonstrated that MTE containing silibinin enhanced osteogenesis by inducing osteoblastogenic biomarker of osteoblast ALP and abrogating RANKL-induced osteoclastogenic biomarker of osteoclast TRAP activity. MTE boosted BMD reduced by uterine deprivation and diminished femoral TRAP activity elevated in OVX mice. Accordingly, MTE works as an osteoporoprotective agent inhibiting osteoclastic bone resorption induced by estrogen deficiency and may serve as a clinical modulator against osteopetrosis and postmenopausal osteoporosis. 

## Figures and Tables

**Table 1:** Effects of milk thistle extract (MTE) and silibinin on growth and toxicity in ovariectomized (OVX) mice.

	Sham control	OVX	OVX-MTE	OVX-silibinin
Initial BW (g)	22.89 ± 2.52	22.92 ± 3.15	22.67 ± 2.04	22.60 ± 1.69
Final BW (g)	23.80 ± 2.31^b^	26.22 ± 2.64^a^	24.86 ± 1.95^ab^	24.18 ± 1.10^ab^
ADG (mg/d)	23.19 ± 3.31^c^	62.65 ± 9.15^a^	56.80 ± 7.39^a^	37.20 ± 6.50^b^
ADFI (g/d)	3.85 ± 0.06	3.86 ± 0.06	4.03 ± 0.06	3.75 ± 0.06
FER	0.59 ± 0.08^c^	1.62 ± 0.24^a^	1.39 ± 0.18^a^	0.99 ± 0.16^b^
Liver weight (g)	1.17 ± 0.08	1.14 ± 0.06	1.14 ± 0.03	1.13 ± 0.05
LW/BW (%)	4.86 ± 0.27^a^	4.38 ± 0.12^b^	4.54 ± 0.19^ab^	4.67 ± 0.25^a^
Serum GOP (IU/L)	50.35 ± 1.94	50.25 ± 1.62	50.16 ± 1.32	48.71 ± 1.69
Serum GPT (IU/L)	22.84 ± 1.31	21.96 ± 1.37	21.77 ± 1.77	21.71 ± 1.32

C57BL/6 female mice (11 weeks of age, 20–25 g) were surgically ovariectomized and orally supplemented with 10 mg/kg MTE and silibinin daily for 8 weeks. BW: body weight; ADG: average daily gain; ADFI: average daily food intake; FER: food efficiency ratio; OVX: ovariectomy; LW: liver weight. Values are means ± SEM of 9 mice. Respective values in the same row not sharing the same superscript differ, *P* < 0.05.

**Table 2:** Osteogenic activity of milk thistle extract (MTE) and silibinin in ovariectomized (OVX) mice.

	Sham control	OVX	OVX-MTE	OVX-silibinin
BMD	74.4 ± 2.0^a^	61.0 ± 0.8^c^	72.5 ± 1.9^a^	67.4 ± 0.4^b^
BMC	17.6 ± 0.4^a^	15.1 ± 0.2^c^	17.2 ± 0.7^a^	16.4 ± 0.2^b^
Bone area	0.237 ± 0.003	0.250 ± 0.004	0.238 ± 0.006	0.244 ± 0.004

C57BL/6 female mice (11 weeks of age, 20–25 g) were ovariectomized and orally supplemented with 10 mg/kg MTE and silibinin daily for 8 weeks. Areal bone mineral density (BMD, mg/cm^2^), bone mineral content (BMC, mg), and bone area (cm^2^) were measured in femur bones by using a PIXImus mouse densitometer. Values are means ± SEM of 9 mice. Respective values in the same row not sharing the same superscript differ, *P* < 0.05.

## Abbreviations

ALP: Alkaline phosphatase
BMD: Bone mineral density
BMC: Bone mineral content
MTE: Milk thistle extract
OPG: Osteoprotegerin
OVX: Ovariectomy
RANK: Receptor activator of nuclear factor-*κ*B
RANKL: Receptor activator of nuclear factor-*κ*B ligand
SBE: Soybean extract
TRAP: Tartrate-resistant acid phosphatase.

## References

[1] Kanis JA (2002). Osteoporosis III: diagnosis of osteoporosis and assessment of fracture risk. *The Lancet*.

[2] Fraser LA, Vogt KN, Adachi JD, Thabane L (2011). Fracture risk associated with continuation versus discontinuation of bisphosphonates after 5 years of therapy in patients with primary osteoporosis: a systematic review and meta-analysis. *Therapeutics and Clinical Risk Management*.

[3] Wensel TM, Iranikhah MM, Wilborn TW (2011). Effects of denosumab on bone mineral density and bone turnover in postmenopausal women. *Pharmacotherapy*.

[4] Beard MK (2012). Bisphosphonate therapy for osteoporosis: combining optimal fracture risk reduction with patient preference. *Current Medical Research and Opinion*.

[5] Silverman S, Christiansen C (2012). Individualizing osteoporosis therapy. *Osteoporos International*.

[6] Åkesson K (2003). New approaches to pharmacological treatment of osteoporosis. *Bulletin of the World Health Organization*.

[7] Putnam SE, Scutt AM, Bicknell K, Priestley CM, Williamson EM (2007). Natural products as alternative treatments for metabolic bone disorders and for maintenance of bone health. *Phytotherapy Research*.

[8] Banu J, Varela E, Fernandes G (2012). Alternative therapies for the prevention and treatment of osteoporosis. *Nutrition Reviews*.

[9] Sunita P, Pattanayak SP (2011). Phytoestrogens in postmenopausal indications: a theoretical perspective. *Pharmacognosy Reviews*.

[10] Al-Anazi AF, Qureshi VF, Javaid K, Qureshi S (2011). Preventive effects of phytoestrogens against postmenopausal osteoporosis as compared to the available therapeutic choices: an overview. *Journal of Natural Science and Biological Medicine*.

[11] Turner JV, Agatonovic-Kustrin S, Glass BD (2007). Molecular aspects of phytoestrogen selective binding at estrogen receptors. *Journal of Pharmaceutical Sciences*.

[12] Tyagi AM, Srivastava K, Sharan K, Yadav D, Maurya R, Singh D (2011). Daidzein prevents the increase in CD4^+^CD28null T cells and B lymphopoesis in ovariectomized mice: a key mechanism for anti-osteoclastogenic effect. *PLoS One*.

[13] Tseng PC, Hou SM, Chen RJ (2011). Resveratrol promotes osteogenesis of human mesenchymal stem cells by upregulating RUNX2 gene expression via the SIRT1/FOXO3A axis. *Journal of Pharmaceutical Sciences*.

[14] Kim JL, Park SH, Jeong D, Nam JS, Kang YH (2012). Osteogenic activity of silymarin through enhancement of alkaline phosphatase and osteocalcin in osteoblasts and tibia-fractured mice. *Experimental Biological Medicine*.

[15] Lu P, Mamiya T, Lu LL (2009). Silibinin prevents amyloid b peptide-induced memory impairment and oxidative stress in mice. *British Journal of Pharmacology*.

[16] Sangeetha N, Aranganathan S, Panneerselvam J, Shanthi P, Rama G, Nalini N (2010). Oral supplementation of silibinin prevents colon carcinogenesis in a long term preclinical model. *European Journal of Pharmacology*.

[17] Kim JL, Kang SW, Kang MK (2012). Osteoblastogenesis and osteoprotection enhanced by flavonolignan silibinin in osteoblasts and osteoclasts. *Journal of Cellular Biochemistry*.

[18] Ryou SH, Kang MS, Kim KI, Kang YH, Kang JS (2012). Effects of green tea or Sasa quelpaertensis bamboo leaves on plasma and liver lipids, erythrocyte Na efflux, and platelet aggregation in ovariectomized rats. *Nutrition Research Practice*.

[19] Akcakaya H, Aroymak A, Gokce S (2007). A quantitative colorimetric method of measuring alkaline phosphatase activity in eukaryotic cell membranes. *Cell Biology International*.

[20] Liu H, Yuan Q, Li CF, Huang TX (2010). Isolation and purification of silychristin, silydianin and taxifolin in the co-products of the silybin refined process from the silymarin by high-speed counter-current chromatography. *Process Biochemistry*.

[21] Lee JI, Hsu BH, Wu D, Barrett JS (2006). Separation and characterization of silybin, isosilybin, silydianin and silychristin in milk thistle extract by liquid chromatography-electrospray tandem mass spectrometry. *Journal of Chromatography A*.

[22] Lee DYW, Liu Y (2003). Molecular structure and stereochemistry of silybin A, silybin B, isosilybin A, and isosilybin B, isolated from Silybum marianum (milk thistle). *Journal of Natural Products*.

[23] Boonen S, Rosenberg E, Claessens F, Vanderschueren D, Papapoulos S (2012). Inhibition of cathepsin K for treatment of osteoporosis. *Current Osteoporosis Reports*.

[24] North American Menopause Society (2012). The 2012 hormone therapy position statement of: the North American Menopause Society. *Menopause*.

[25] Sutton EE, Riche DM (2012). Denosumab, a RANK ligand inhibitor, for postmenopausal women with osteoporosis. *Annals of Pharmacotherapy*.

[26] Wu J, Wang X, Chiba H (2004). Combined intervention of soy isoflavone and moderate exercise prevents body fat elevation and bone loss in ovariectomized mice. *Metabolism: Clinical and Experimental*.

[27] Sacco SM, Jiang JMY, Reza-López S, Ma DWL, Thompson LU, Ward WE (2009). Flaxseed combined with low-dose estrogen therapy preserves bone tissue in ovariectomized rats. *Menopause*.

[28] Schümann J, Prockl J, Kiemer AK, Vollmar AM, Bang R, Tiegs G (2003). Silibinin protects mice from T cell-dependent liver injury. *Journal of Hepatology*.

[29] Boyle WJ, Simonet WS, Lacey DL (2003). Osteoclast differentiation and activation. *Nature*.

